# Genome-wide association study of 23 flowering phenology traits and 4 floral agronomic traits in tree peony (*Paeonia* section *Moutan* DC.) reveals five genes known to regulate flowering time

**DOI:** 10.1093/hr/uhac263

**Published:** 2022-12-02

**Authors:** Yuying Li, Lili Guo, Zhanying Wang, Dehui Zhao, Dalong Guo, John E. Carlson, Weilun Yin, Xiaogai Hou

**Affiliations:** College of Agronomy/College of Tree Peony, Henan University of Science and Technology, Luoyang, Henan, 471023, China; College of Agronomy/College of Tree Peony, Henan University of Science and Technology, Luoyang, Henan, 471023, China; Luoyang Academy of Agricultural and Forestry Sciences, Luoyang, Henan, 471000, China; College of Agronomy/College of Tree Peony, Henan University of Science and Technology, Luoyang, Henan, 471023, China; College of Forestry, Henan University of Science and Technology, Luoyang, Henan, 471023, China; Department of Ecosystem Science and Management, Pennsylvania State University, University Park, PA 16802, USA; College of Biological Sciences and Technology, Beijing Forestry University, Beijing 100083, China; College of Agronomy/College of Tree Peony, Henan University of Science and Technology, Luoyang, Henan, 471023, China

## Abstract

Tree peony is a unique traditional flower in China, with large, fragrant, and colorful flowers. However, a relatively short and concentrated flowering period limits the applications and production of tree peony. A genome-wide association study (GWAS) was conducted to accelerate molecular breeding for the improvement of flowering phenology traits and ornamental phenotypes in tree peony. A diverse panel of 451 tree peony accessions was phenotyped for 23 flowering phenology traits and 4 floral agronomic traits over 3 years. Genotyping by sequencing (GBS) was used to obtain a large number of genome-wide single-nucleotide polymorphisms (SNPs) (107 050) for the panel genotypes, and 1047 candidate genes were identified by association mapping. Eighty-two related genes were observed during at least 2 years for flowering, and seven SNPs repeatedly identified for multiple flowering phenology traits over multiple years were highly significantly associated with five genes known to regulate flowering time. We validated the temporal expression profiles of these candidate genes and highlighted their possible roles in the regulation of flower bud differentiation and flowering time in tree peony. This study shows that GWAS based on GBS can be used to identify the genetic determinants of complex traits in tree peony. The results expand our understanding of flowering time control in perennial woody plants. Identification of markers closely related to these flowering phenology traits can be used in tree peony breeding programs for important agronomic traits.

## Introduction

Tree peony (*Paeonia* section *Moutan* DC.) is a famous traditional flower that originated in China and is the first candidate for China’s national flower, with striking ornamental value [1, 2]. It has been termed the ‘king of flowers’ for its large and various flower forms and rich, bright colors, which symbolize happiness, wealth, and prosperity in Chinese culture [3, 4]. Tree peony is also an emerging woody oil crop with high economic value [5]. Flowering is a key ornamental character of the tree peony, but its flowering period is short and relatively uniform. Under natural conditions, it takes only 50–60 days from budding to fading; the flowering period is 3–5 days for a single flower and 10–15 days for a colony of plants. Flowering characteristics, particularly flowering period, have limited the commercial development of tree peony. Although there are many varieties of tree peony, most are middle-flowering varieties, and the proportions of early- and late-flowering varieties are small.

Early or delayed flowering has been achieved in most ornamental plants through research on the regulation of flowering phenology, thus enriching ornamental flower varieties to meet different ornamental and economic needs [6–8]. Flowering regulation is therefore especially important in tree peony breeding. Mostresearchers are eager to break through the limitations on tree peony flowering period by crossbreeding, but the breeding cycle is long, and trait separation is difficult to control [9, 10]. Molecularstudies on tree peony started relatively late but developed rapidly, focusing mainly on the development of molecular markers [9–11], second- or third-generation high-throughput sequencing [2, 3, 12],construction of genetic maps [11, 13], and cloning of related genes[14–16].

With the rapid development of next-generation sequencing (NGS) in recent years, 69 ornamental plant genomes have beensequenced [17]. Genome-wide association study (GWAS) is a newtechnique for genome-wide control or association analysis usingmillions of genomic single-nucleotide polymorphisms (SNPs) asmolecular markers and to discover genetic variations affecting complex traits [18–21]. At present, the method has been applied to screen and identify major genes for economically valuable traits [22, 23]. Flowering traits of some plants have also been evaluated by GWAS in other studies. Genomic regions associated with flowering period and petal, stigma, calyx, and bud colors have been identified by GWAS in *Prunus mume* [17, 24]. Combined with F-one association mapping, 61 SNPs related to altitude were found in 22 environments and were also related to maize flowering time [25]. GWAS of flowering phenology in *Brassica napus* identified regions that overlapped with previously known quantitative trait loci (QTLs), and significant correlations were found between flowering time and *Bna.CCA1*, *Bna.FT*, *Bna.FUL*, and *BnVIN3-C03* [26–29]. GWAS revealed seven reliable SNPs associated with cowpea flowering time, including SNPs in *FT*, *GI*, *CRY2*, *LSH3*, *UGT87A2*, *LIF2*, and *HTA9*, which explained 8%–12% of the phenotypic variation [30]. The first draft genome of tree peony has recently been published [3], enabling further research on the molecular mechanisms of tree peony flowering regulation using GWAS.

The genetic regulation of flowering has been studied most extensively in model plants and many crops [17, 31–33] and is known to involve multiple pathways. These mainly include the photoperiodic, vernalization, gibberellin, and autonomic pathways, followed by the carbohydrate induction and floral inhibition pathways. In recent years, the aging pathway has also been shown to regulate the floral transition [34, 35]. These genetic pathways regulate flowering through positive or negative regulation of genes and/or transcription factors, including *FLOWERING LOCUS C* (*FLC*), *FRIGIDA* (*FRI*), and *VERNALIZATION INSENSITIVE 3* (*VIN3*), which sense low-temperature signals [26, 36] ; *APETALA1* (*AP1*), *FRUITFUL* (*FUL*), and *CAULIFLOWER* (*CAL*), which regulate floral organ development [37, 38]; *FLOWERING LOCUS T* (*FT*), *LEAFY* (*LFY*), *CONSTANS* (*CO*), and *SUPPRESSOR OF OVEREXPRESSION OF CO1* (*SOC1*), which regulate flower formation by the long-day pathway [35, 39, 40]; and genes related to flower bud dormancy release, such as *EARLY BUD BREAK1* (*EBB1*) and *DORMANCY ASSOCIATED MADS-BOX* (*DAM*) [41, 42]. Furthermore, microRNAs (miRNAs) also modulate flowering by regulating the expression of flowering-related target genes, and multiple miRNAs play different roles in flower bud differentiation and flower induction. Overexpression of *miRNA172* alleviated the flowering delay caused by *TOE1* expression [43], whereas overexpression of *miRNA159* delayed flowering, accompanied by decreased expression levels of *MYB33* and *LFY* [44]. The interaction network among the genes and gene products that regulate flowering time is extremely complex. Although research on this network has made fundamental progress in molecular and developmental biology, it has focused mainly on model plants. The molecular mechanisms of flowering in many woody ornamental plants have not been thoroughly studied, and studies have usually been carried out with only limited genomic information [13].

Although GWAS has been widely used in many plants, its role in tree peony is unclear. Because of a limited number of markers and the complex tree peony genome, it has been difficult for us to accurately identify candidate regions for target traits and to understand the development and floral formation mechanism of tree peony flowers. To more fully understand the molecular mechanisms that regulate flowering time in tree peony, we recorded flowering phenology characteristics of 451 peony varieties over 3 years. Genotyping by sequencing (GBS) and GWAS approaches were used to locate important genetic regions for flowering regulation in tree peony. The results provide a theoretical basis for artificial regulation of flower development in production practice and for breeding new varieties with different flowering dates; they also provide a reference for the study of flowering mechanisms in other woody plants.

## Results

### General flowering phenology observations

**Figure 1:**
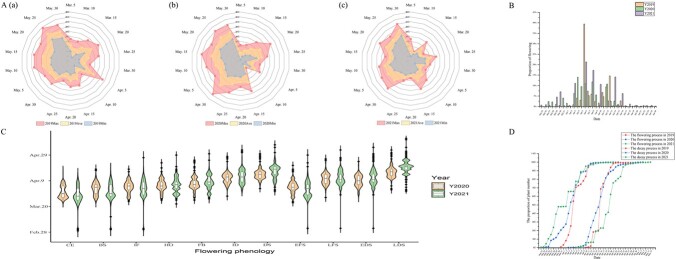
Phenotypic analysis of flower phenology traits. (A) Temperature distribution during the observation period in 2019 (a), 2020 (b), and 2021 (c). (B) Flowering proportion through time for 2019–21. (C) Violin plots of flowering phenology traits in 2020 and 2021. (D) Flowering and decay processes of tree peony varieties in three years.

The temperature differences between the highest and lowest temperatures were relatively stable during the survey period in 2019, 2020, and 2021, mainly concentrated from 25 to 27°C, except in April 2020, when a difference of 32°C was observed (Fig. 1A). By observing the flowering phenology of tree peonies, we found that all flowering events were completed in March and April every year (Fig. 1B and C) and that flowering phenology was closely related to temperature changes. In 2019, the early full blooming stage of the collection of 596 ornamental tree peonies began on 31 March, the full blooming stage on 4 April, the decay stage on 7 April, and the late decay stage on 10 April; the complete flowering duration was 33 days. In 2020, the tree peonies entered the early full blooming stage on 23 March and the late decay stage on 3 April; the complete flowering duration was 41 days. In 2021, the tree peonies entered the early full blooming stage on 23 March, the full blooming stage on 26 March, and the late decay stage on 6 April; the complete flowering duration was 50 days (Fig. 1D).

Significant variation (*P* < .001) in flowering phenology-related traits was observed in the 596 tree peony varieties from 2019 to 2021 (Supplementary Data Table S2). The timing of the color-exposure stage (TCE) for each tree peony variety was 21.70 ± 0.20 days, the timing of the full blooming stage (TFB) was 29.70 ± 0.20 days, and the timing of the decay stage (TDS) was 40.14 ± 0.17 days (Supplementary Data Table S2). The duration of the full blooming stage (DFB) and the duration of the decay stage (DDS) were 12.08 ± 0.20 and 20.13 ± 0.20 days, respectively (Supplementary Data Table S2). Based on the onset of full bloom in 2019–21, the 596 tree peony varieties could be divided into 4 extremely early-flowering varieties, 152 early-flowering varieties, 350 middle-flowering varieties, 86 late-flowering varieties, and 4 extremely late-flowering varieties (Supplementary Data Table S1).

The coefficients of variation (CVs) of 30 phenotypic traits ranged from 9.85% to 48.37%, with an average of 24.91%. The CV of flower height (FH) was the lowest, and that of the number of flowers (NF) was 47.41%, second only to that of duration of the early full blooming period (DEFS) (Supplementary Data Table S2). Compared with the flowering phenology timing-related traits (average CV 15.76%), the flowering phenology duration-related traits showed greater variation (average CV 35.68%). The average CV of floral traits [NF, FH, flower diameter (FD), and pedicel length (PL)] was 22.70%, and that of plant traits [plant height (PH), crown diameter (CD), and crown shade size (CSS)] was 25.35%. There were significant differences in traits among genotypes and years (*P* < .05). Further analysis showed that the diversity index of duration-related traits ranged from 6.29 to 6.37, with an average of 6.33. The maximum diversity index among the flowering phenology timing-related traits was 6.39 for the timing of the late decay stage (TLDS), the minimum was 6.37 for TCE, and the average was 6.38, higher than that of flowering phenology duration-related traits.

**Figure 2:**
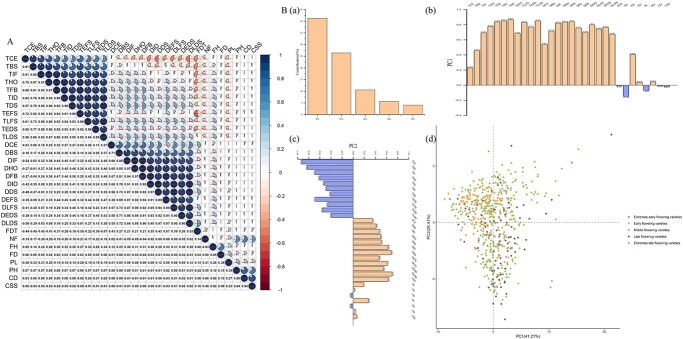
Statistical analysis of 30 phenotypic traits of 596 tree peony varieties. (A) Correlation analysis. ^*^P < .05, ^**^P < .01, ^***^P < .001. (B) PCA. (a) Percentage of variance explained by the first six components in the analysis. (b) and (c) Eigenvectors for the variables in the first two components. (d) Scatterplot showing the distribution of tree peony varieties along the first two principal components.

Several significant correlations were observed between traits (Fig. 2A, Supplementary Data S1). The 11 flowering phenology timing-related traits were positively correlated with one another (average correlation coefficient 0.85, *P* < .001). The 11 flowering phenology duration-related traits were also positively correlated with one another (average correlation coefficient 0.83, *P* < .001). Among the 30 total traits, 24 were significantly correlated with flowering duration time (FDT). Among the flower traits, there was no correlation between NF and FH and a significant positive correlation between NF and FD (*P* < .05); the other traits showed extremely significant positive correlations (*P* < .001). There were extremely significant positive correlations among PH, CD, and CSS (0.80, 0.64, and 0.85; *P* < .001).

Five principal components (eigenvalues >1) were extracted from the 30 traits, with a cumulative contribution rate of 87.92% and an eigenvalue total of 26.38; the contribution rate of the first principal component (PC1) was 41.21% (Fig. 2Ba, Supplementary Data S2). The matrix values of each trait obtained by principal component analysis (PCA) reflected the distribution of phenotypic traits along each principal component. The characteristic value of PC1 was 12.49, covering 10 flowering phenology timing-related traits and 8 flowering phenology duration-related traits. Therefore, it could be called the flowering phenology factor (Fig. 2Bb, Supplementary Data S2). The contribution rate of the second principal component (PC2) was 26.41%, and the main representative indexes were duration of the late decay stage (DLDS) and FDT (Fig. 2Bc, Supplementary Data S2). The contribution rate of the third principal component was 10.63%, and the most influential traits were NF, PH, CD, and CSS. The characteristic values of the third principal component were all >0.70, and it could therefore be considered a plant trait factor. The fourth principal component was strongly influenced by FH, FD, and PL, whose characteristic values were 0.76, 0.81 and 0.46, respectively, and it could therefore be considered a flower trait factor. The representative indexes of the fifth principal component were duration of the color-exposure stage (DCE) and TCE (Supplementary Data S2). The 596 cultivated tree peony varieties were evenly distributed along the coordinate axes of PC1 and PC2 (Fig. 2Bd), and no obvious hierarchical structure was formed.

### Phenotypic diversity of major traits in the GWAS population

To prevent spurious associations due to population stratification, we did not measure all agronomic traits in 596 varieties and used only the phenotypes obtained from 451 varieties. The maximum value, minimum value, mean value, standard error, CV, and diversity index of 30 traits from the 451 varieties were consistent with those of the original 596 varieties, and they showed good uniformity and abundance (Supplementary Data Fig. S1, Supplementary Data Table S2). The 451 varieties had a higher coincidence rate of range (CR = 93.33%), variable rate of coefficient of variation (VR = 94.34%), variance difference percentage (VD = 40.00%), and phenotype retention ratio (PRR = 100%), indicating that the genetic variation of each trait in the 596 original varieties was present in the 451 selected varieties and was evenly distributed with small genetic redundancy. The 451 varieties selected for subsequent GWAS analysis included 4 extremely early-flowering varieties, 121 early-flowering varieties, 280 middle-flowering varieties, 44 late-flowering varieties, and 2 extremely late-flowering varieties (Supplementary Data Table S1).

There was a high degree of phenotypic variation among the 451 tree peony varieties over years (*P* < .001), and the CVs of the measured traits ranged from 9.56% to 44.34%, with an average of 23.15% (Supplementary Data Table S2). The average CV of the four flower traits was 21.18%. Flowering phenology statistics showed that the FDT of the 451 varieties ranged from 8.67 to 18.33 days. The CV of the flowering phenology duration-related traits (32.55%) was greater than that of the flowering phenology timing-related traits (15.28%). The timing and duration of full blooming (FB) also differed among varieties. The earliest TFB was 17.00 days and the latest was 47.00 days; the shortest DFB was 3.50 days, whereas the longest was 27.00 days. The flowering phenology timing-related traits of the different varieties showed high consistency, with a correlation coefficient **r*^2^* > .62, *P* < .001 (Supplementary Data Fig. S3, Supplementary Data S3). There was a weak correlation between DCE and duration of DEFS (correlation coefficient 0.31, *P* < .001), and the correlation between DCE and duration of the early decay stage (DEDS) was relatively weak (correlation coefficient 0.46, *P* < .001). In addition, the flowering phenology duration-related traits were highly correlated with the flowering phenology timing-related traits (*r*^2^ > 0.51, *P* < .001). Notably, FDT was significantly negatively correlated with the flowering phenology timing-related traits but positively correlated with the flowering phenology duration-related traits (Supplementary Data Fig. S3, Supplementary Data S3). Plant traits were only weakly correlated with flowering phenology and floral traits. Therefore, only 23 flowering phenology-related traits and 4 floral traits were considered in the subsequent association analysis (Supplementary Data Fig. S3, Supplementary Data S3). The 27 phenotypic traits analyzed in the 451 tree peony varieties showed continuous variation and basically corresponded to a normal distribution (Supplementary Data Fig. S2).

### Characterization and distribution of SNPs in the tree peony genome

Approximately 1321.5 Gb of clean sequencing reads were generated from the 451 tree peony varieties, with an average of 2.9 Gb per sample (Supplementary Data Table S3). About 95.84% of the reads were successfully mapped to the tree peony reference genome [3] with an average depth of 9.87× (Supplementary Data Table S3). A total of 45 236 236 SNPs were initially identified among the 451 tree peony varieties using GATK version 2.4, and 107 050 SNPs were retained after filtering. Most of the retained SNPs were located in intergenic regions (91.19%) or intronic regions (5.72%), whereas 671 were found in exons, including 18 putatively damaging SNPs (stop codon gain/loss). Among the SNPs in coding regions (CDSs), 47.69% were synonymous and 49.63% non-synonymous. In addition, 77 were located in 3′ UTRs, 172 were located in 5′ UTRs, and the remaining 811 had no annotation information. The genome-wide transition/transversion (Ts/Tv) ratio was 2.076, and the number of SNPs in transversion (34 794) was lower than that in transition (72 256) (Supplementary Data Table S4).

### Population structure of tree peony germplasm

**Figure 3:**
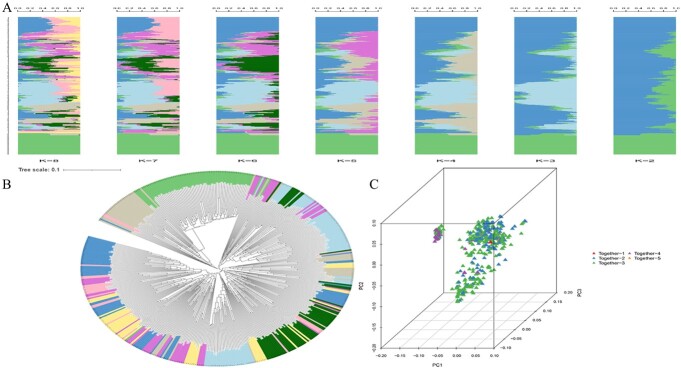
Genetic structure of 451 tree peony varieties. (A) Population structure analysis. (B) Phylogenetic tree, with colors filled according to the population structure. (C) PCA.

We used STRUCTURE v2.3.4 to analyze population structure with 107 050 high-quality SNPs from 451 tree peony varieties to estimate individual ancestry and mixing ratio. When *K* = 8, the overall structure was highly mixed (Fig. 3A). The genetic differentiation of population genotypes was analyzed further, and the phylogenetic relationships among the genotypes were calculated. Based on the comparison of color classification, the phylogenetic tree, and structural analysis, most germplasms in the same group in the population structure also showed close relationships in the phylogenetic tree analysis (Fig. 3B). PCA showed no significant population stratification among extremely early-, early-, middle-, late-, and extremely late-flowering varieties and also revealed the diversity of the tree peony genotypes (Fig. 3C).

### GWAS of flowering phenology

Based on *P* < 9.34E−04, GWAS detected 3816 significant SNPs associations with 27 traits over 3 years, and the number of SNPs associated with each trait ranged from 131 to 439 (Supplementary Data Table S5). The quantile–quantile plots showed that the mixed linear model (MLM) made a reasonable correlation in controlling for the overall false-positive association that may result from underlying population structure (Supplementary Data Figs S4–S7). The SNPs were annotated in 1047 genes: 187, 519, and 522 genes in 2019, 2020, and 2021, respectively, and 176 genes were associated with different traits in at least 2 years (Supplementary Data Tables S6–S8). One hundred and seven genes were repeatedly identified with a single trait in at least 2 years, including one gene for duration of the blooming stage (DBS), duration of the late full blooming stage (DLFS), DLDS, and FDT, 2 for duration of the initial flowering stage (DIF), duration of the half opening stage (DHO), and FH, 3 for DCE, timing of the early full blooming stage (TEFS), and FD, 4 for TCE, timing of the late full blooming stage (TLFS), and PL, 5 for timing of the blooming stage (TBS), timing of the initial flowering stage (TIF), TDS, and TLDS, 7 for timing of the half opening stage (THO), 9 for TFB, 11 for timing of the initial decay stage (TID), 12 for timing of the early decay stage (TEDS), and 17 for NF. However, no interannual repetition was detected for DFB, timing of the initial decay stage (DID), DDS, DEFS, and DEDS (Supplementary Data Table S5).

**Figure 4:**
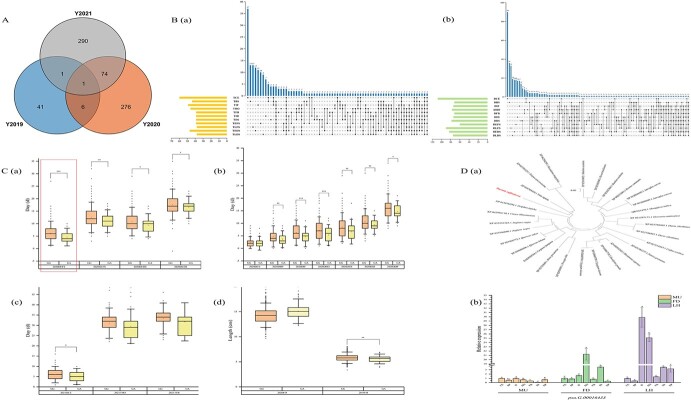
Profile of loci associated with flowering-related traits of the tree peony population over 3 years. (A) Venn diagram of loci associated with 23 traits over 3 years (2019–21). (B) (a) Bar chart of the associations of 11 flowering phenology timing-related traits and their interactive upset plot. (b) Bar chart of the associations of 11 flowering phenology duration-related traits and their interactive upset plot. (C) The allelic effect at 059892F:42007 for the significantly associated target traits in different years (*P* < .05) in the association panel. (D) The candidate gene *PoFY* was analyzed by evolutionary tree (a) and its expression profile during flowering (b).

A total of 2446 SNPs related to 23 flowering phenology traits were detected in 2019–21 (169 significantly associated SNPs in 2019, 1277 in 2020, and 1181 in 2021, In addition, 179 SNPs were repeatedly associated for two consecutive years, and one SNP was simultaneously associated for three years. *P* < .001). These SNPs were annotated to 49, 357, and 366 genes, respectively, 82 of which were annotated in at least 2 years (Fig. 4A, Supplementary Data Tables S6 and S7). A number of annotated genes overlapped significantly among multiple flowering phenology traits. Eighty genes were significantly associated with at least two traits related to flowering phenology timing, and three genes (*psu.G*.*00032476*, *psu.G.00027564*, and *psu.G.00012770*) were significantly associated with all 11 flowering phenology timing-related traits. *Psu.G.00030531*, *psu.G.00001085*, *psu.G.00001060*, and *psu.G.00019234* were associated with at least 10 flowering phenology timing-related traits (TBS, TIF, THO, TFB, TID, TDS, TEFS, TLFS, TEDS, TLDS) (Fig. 4Ba, Supplementary Data Table S8). One hundred and sixty-four genes were also significantly associated with at least two traits related to flowering phenology duration. Among them, *psu.G.00011319* (DCE, DBS, DIF, DHO, DFB, DID, DEFS, DLFS, DEDS), *psu.G.00026344* (DBS, DIF, DHO, DFB, DID, DDS, DEFS, DEDS, DLDS), *psu.G.00003074* (DIF, DHO, DFB, DID, DDS, DEFS, DLFS, DEDS, DLDS), and *psu.G.00027268* (DIF, DHO, DFB, DID, DDS, DEFS, DLFS, DEDS, DLDS) were repeatedly associated with nine flowering phenology duration-related traits (Fig. 4Bb, Supplementary Data Table S8). A total of 233 SNPs were associated with multiple flowering phenology traits in 3 years, 67.81% of which (158) were located in intergenic regions. Forty-nine were located in intronic regions, 11 in exons, and the remaining 6, 6, 2, and 1 in downstream regions, upstream regions, 5′ UTRs, and 3′ UTRs, respectively (Supplementary Data Table S8).

GWAS analysis of TCE in 2020 and 2021 showed a significant peak on the Manhattan map, which was located at 153530F:9938 (*P* = 9.47329E−06), corresponding to *psu.G.00027564* in the tree peony genome. This gene was primarily annotated as Receptor-like protein kinase (Supplementary Data Fig. S5Aa and b, Supplementary Data Tables S6 and S7). In the GWAS analysis of TBS, 020514F:1844 (*P* = 6.04185E−06) was found to be significantly associated with TBS in 2021. This locus was not annotated on the reference genome of tree peony, so it was speculated that it might be a new locus associated with TBS (Supplementary Data Fig. S5Bb, Supplementary Data Table S6). Furthermore, an extremely significant peak was found in the Manhattan map of the THO (2020) association analysis (000416F:34642, *P* = 5.26246E−08) (Supplementary Data Fig. S5Da, Supplementary Data Table S6). In 2021, it was found that the locus located in 089531F:20262 (*P* = 6.27495E−06) was the most significant associated with THO (Supplementary Data Fig. S5Db, Supplementary Data Table S6), and there was no corresponding annotation information for both of them. Therefore, it was inferred that these two loci were very likely to be new loci related to THO, and their detailed functional information needs to be further studied. Similarly, loci closely associated with the corresponding traits were captured in the GWAS analysis of other flowering phenology timing-related traits and the flowering phenology duration-related traits, and a large number of new loci are also not annotated at present.

Homologs of some identified genes were previously reported to be associated with flowering in other plants. A locus significantly associated with DEFS was identified in 2020; it was located at 059892F:42007 (*P* = 1.028380E−02) and was annotated as *psu.G.00016433* (Supplementary Data Fig. S6h). This gene was highly homologous to *FY* from *Arabidopsis thaliana*. *FY* plays a role in flowering time regulation by reducing the mRNA level of *FLC*, and it is necessary for negative autoregulation of *FCA* expression. Genotype analysis of this locus revealed that there were 399 individuals with the GG genotype and 47 individuals with the heterozygous mutation genotype (GA) in the population. Further analysis showed that DEFS was significantly longer in GG individuals than in GA individuals by 1.40 days (*P* < .01) in 2020 (Fig. 4Ca, highlighted with a red box). Individuals with the GG genotype had significantly higher (*P* < .05) flowering phenology-related trait values than those with the GA genotype in different years (Fig. 4Ca–d). In addition, phenotypic differentiation in flower traits was also significantly associated with this locus. FH was significantly higher by 0.23 cm in the GG genotype compared with the GA genotype in 2019 (*P* < .01) (Fig. 4Cd), and FD was significantly shorter in the GG genotype than the GA genotype in 2020 (*P* < .01) (Fig. 4Cd). To verify the variation at this locus, a 1680-bp sequence was cloned and named *PoFY*. The open reading frame (ORF) of *PoFY* was 1629 bp, encoding 542 amino acids. Further analysis showed a high degree of similarity (89.27%) with its homolog in *Vitis vinifera* (Fig. 4Da). The expression of *PoFY* was measured in seven flower developmental periods [color exposure (CE), blooming (BS), initial flowering (IF), half opening (HO), full blooming (FB), initial decay (ID), and decay (DS)] in an early-flowering mutant and wild *Paeonia ostii* ‘Fengdan’ and *Paeonia suffruticosa* ‘Lianhe’ (MU, FD, and LH for short). Quantitative real-time PCR (qRT–PCR) results showed that *PoFY* expression was generally low in MU; it decreased gradually from flower development to FB, then increased gradually during the decay process. By contrast, the *PoFY* expression levels of FD and LH showed a trend of first increasing and then decreasing at FB, then decreasing during the decay process (Fig. 4Db). These results suggested that *PoFY* was a candidate gene for flowering time regulation.

**Figure 5:**
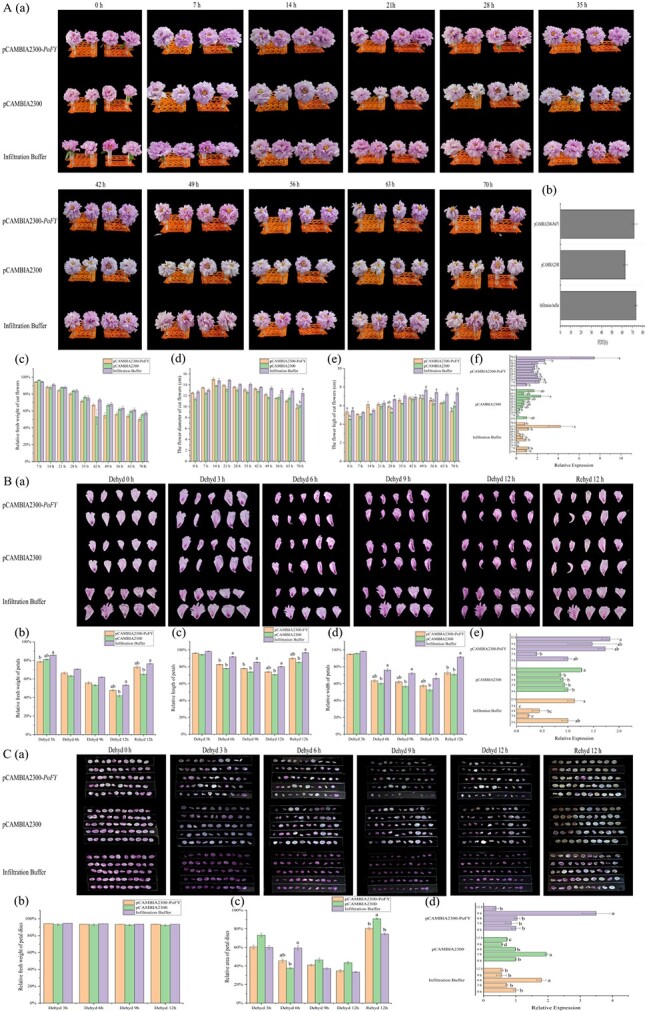
Transient expression of *PoFY* was overexpressed in fresh cut flowers, petals, and petal disks of tree peony. (A) Overexpression infection in fresh cut flowers of tree peony. The phenotype (a), FDT (b), relative fresh weight (c), flower diameter (d), flower height (e), and relative expression (e) of cut flowers were analyzed. (B) Tree peony petals were infiltrated with *A. tumefaciens* containing pCAMBIA2300-*PoFY*, pCAMBIA2300 and infiltration buffer for dehydration (0, 3, 6, 9, 12 hours) and rehydration for 12 hours. The phenotype of petals (a), relative petal fresh weight (b), relative length (c), relative width (d), and relative expression (e) were analyzed. (C) Phenotype and recovery of *PoFY*-overexpressing petal disks. The phenotype of petal disks (a), relative fresh weight (b), relative area (c), and relative expression (e) were analyzed. Error bars represent the standard error. Different lower-case letters indicate significant differences at *P* < .05. Dehyd, dehydration; Rehyd, rehydration.

In order to detect the possible role of *PoFY* in the regulation of tree peony flowering, we overexpressed *PoFY* in *P. suffruticosa* ‘Luoyanghong’ using a transient expression technique. As expected, for the infection process of *P. suffruticosa* ‘Luoyanghong’ with fresh cut flowers, it revealed that the fresh cut flowers infected with pCAMBIA2300-*PoFY* overexpression vector reached the FB stage 7 hours earlier than flowers infected with empty vector (Fig. 5Aa, Supplementary Data Table S9). Moreover, the time from fully opened flower to abscission of all petals in the cut flowers infected with pCAMBIA2300-*PoFY* overexpression vector was 71.75 ± 2.63 hours, thus longer than that in flowers infected with pCAMBIA2300 empty vector control (63.00 ± 2.02 hours). The FDT was prolonged for 8.75 hours and not statistically significantly different from all infected cut flowers of *P. suffruticosa* ‘Luoyanghong’ (*P* > .05) (Fig. 5Aa and b, Supplementary Data Table S9). Further attention was paid to the change of relative fresh weight among treatments after infection. In the flowering process of cut flowers of *P. suffruticosa* ‘Luoyanghong’, there was no significant difference in relative fresh weight of cut flowers among all treatments (*P* > .05) (Fig. 5Ac). At the early stage of the flowering process, the cut flowers infected with pCAMBIA2300-*PoFY* overexpression vector showed larger flower diameter (Fig. 5Ad, 0–49 hours) and flower height (Fig. 5Ae, 0–56 hours) than those infected with pCAMBIA2300 empty vector control. The expression level of *PoFY* in the fresh cut flowers of *P. suffruticosa* ‘Luoyanghong’ was determined. It was found that the expression level of *PoFY* in the petals infected with overexpression vector reached its highest level at 70 hours after transient expression (Fig. 5Af).

**Figure 6:**
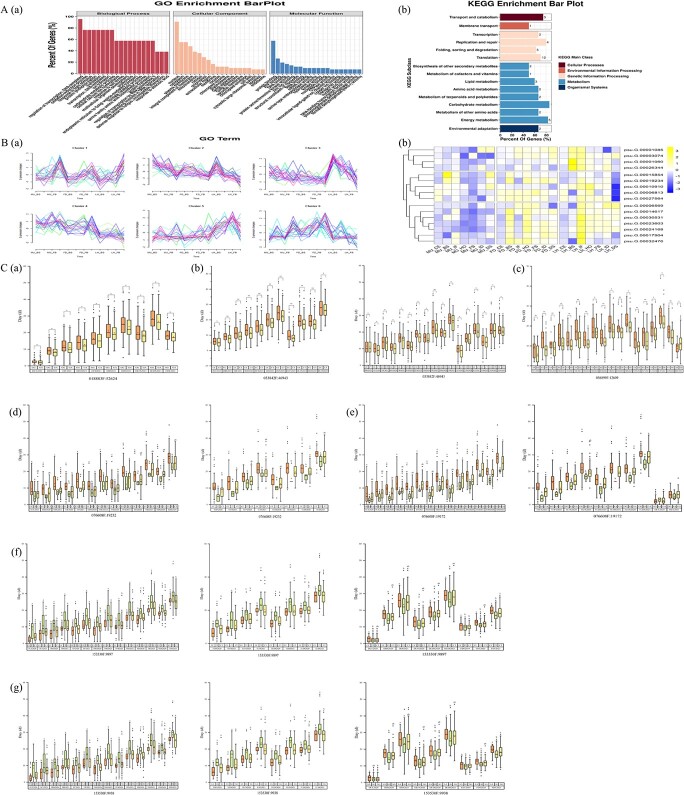
Integration of GWAS analysis with transcriptome sequencing. (A) GO (a) and KEGG (b) enrichment of 200 candidate genes. (B) Cluster profiles of differential gene expression (a) and expression profiles of selected gene candidates during the flowering process (b). (C) Allelic effects at seven candidate loci for the significantly associated target traits in different years (*P* < .05).

To further understand the effect of *PoFY* overexpression on tree peony flowers, we dehydrated the infected petals of *P. suffruticosa* ‘Luoyanghong’. Shrinking of the dehydrated petals was used to illustrate the water loss of the petals in the decaying process of tree peony. During dehydration for 6–12 hours, the relative fresh weight of petals infected with pCAMBIA2300-*PoFY* overexpression vector was larger than that of petals infected with pCAMBIA2300 empty vector, but there was no significant difference (*P* > .05) (Fig. 5Ba and b). After 12 hours of rehydration, the relative fresh weight of petals infected with pCAMBIA2300-*PoFY* overexpression vector was 72.70% ± 1.38% of its initial value, which was 7.57% heavier than that of the empty vector control (65.14 ± 1.02%) (Fig. 5Bb). The same phenotypic difference was observed in relative petal length (Fig. 5Ba and c). After rehydration for 12 hours, the relative length of petals recovered in all treatments. The relative length of petals infected with pCAMBIA2300-*PoFY* overexpression vector returned to 89.97 ± 0.86% of their initial value, which was still significantly longer than that of the empty vector control (*P* < .05) (Fig. 5Ba and c). Moreover, in the process of dehydration, the relative width of petals infected with *Agrobacterium tumefaciens* (regardless of overexpression vector or empty vector) was significantly smaller than that of petals treated with the infiltration buffer control (*P* < .05) (Fig. 5Ba and d). During the rehydration process, the relative width of petals infected with pCAMBIA2300-*PoFY* overexpression vector returned to 72.93 ± 2.17% of their initial value, and the relative width of petals infected with pCAMBIA2300 empty vector returned to 71.01 ± 1.54% of their initial value. However, the relative width of petals treated with the infiltration buffer control returned to 91.50 ± 0.97% of their initial value, which was significantly higher than that of petals infected with *A. tumefaciens* (*P* < .05) (Fig. 6Ba and d). In conclusion, the petals infected with pCAMBIA2300-*PoFY* overexpression vector during dehydration had a lower degree of contraction compared with the petals of empty vector control, which was consistent with the phenotype obtained by prolonging the flowering period after the overexpression of *PoFY* in cut flowers. qRT–PCR showed that the expression of *PoFY* in the petals infected with pCAMBIA2300-*PoFY* overexpression vector after 6 hours of dehydration was indeed higher than that of the control (pCAMBIA2300 empty vector and infiltration buffer control), and expression was significantly higher at 12 hours after dehydration (Fig. 5Be).

The effects of *PoFY* overexpression and dehydration were also tested on petal disks. In the process of dehydration, the relative fresh weight of petal disks infected with pCAMBIA2300-*PoFY* overexpression vector was slightly higher than that of those infected with pCAMBIA2300 empty vector control, but there was no significant difference (*P* > .05) (Fig. 5Ca and b). After rehydration for 12 hours, all petal disks returned to 92.22 ± 1.19%–93.55 ± 0.11% of their initial values (Fig. 5Ca and b). The relative area of petal disks showed a very different pattern (Fig. 5Ca and c). After 6 hours of dehydration, the relative area of petal disks infected with pCAMBIA2300-*PoFY* overexpression vector was 45.60 ± 2.54% of their initial value, which was higher than that of petal disks infected with pCAMBIA2300 empty vector control (37.62 ± 0.83%), but there was no significant difference (*P* > .05) (Fig. 5Ca and c). After rehydration for 12 hours, petal disks infected with pCAMBIA2300 empty vector control returned to 91.02 ± 0.73% of their initial value, which was significantly higher than that of disks infected with the overexpression vector (80.46 ± 1.10%) and those infected with the infiltration buffer control (74.56 ± 1.01%) (*P* < .05) (Fig. 5Cc). The expression of *PoFY* in the overexpressing infected petal disks reached the peak at 9 hours after dehydration (Fig. 5Cd).

### Stable major candidate genomic regions

A total of 200 candidate genes were significantly associated with multiple flowering phenology traits of tree peony (Supplementary Data Table S8). Functional enrichment analysis indicated that these candidate genes were primarily involved in biological processes including flower development (GO:0009908), ethylene-activated signaling pathway (GO:0009873), multicellular organism development (GO:0007275), response to abscisic acid (GO:0009737), and positive regulation of flower development (GO:0009911). In particular, GO:0009738, GO:0009742, and GO:0009753 were associated with hormone-mediated regulatory pathways (Fig. 6Aa, Supplementary Data Table S10). In addition, 52 KEGG-annotated genes were enriched in the carbohydrate metabolism pathway and lipid metabolism pathway, which are closely related to plant flowering induction (Fig. 6Ab, Supplementary Data Table S11). To validate the differential expression of candidate genes significantly associated with flowering phenology-related phenotypes, RNA-seq analysis was performed at four flower developmental periods (BS, IF, FB, and DS) on three representative varieties (MU, FD, and LH; three biological replicates each) with distinct flowering phenology phenotypes. One hundred forty-six significantly differentially expressed genes (DEGs, *P* < 0.05) were identified (Supplementary Data Fig. S7A). Subsequently, we used qRT–PCR to validate the expression profiles of the five randomly screened DEGs and found that the relative expression profiles were consistent with the RNA-seq results of cultivars MU, FD, and LH, indicating the reliability of the data obtained by RNA-seq (Supplementary Data Fig. S7B).

Based on phenotype, all DEGs could be divided into six clusters (Fig. 6Ba). There was a strong correlation between clusters 1 and 2 and tree peony flower development. Sixteen genes closely related to several flowering phenology traits and annotated as related to flowering regulation were selected and analyzed. SNPs (002009F:158561, 002057F:107636, 006960F:97359, 076608F:19232, 076608F:19172, 138984F:12031, 153530F:9938, 153530F:9897, 197535F:3398, 246539F:5389, and 246539F:5280) in *psu.G.00001060*, *psu.G.00001085*, *psu.G.00003074*, *psu.G.00019234*, *psu.G.00026344*, *psu.G.00027564*, *psu.G.00030531*, and *psu.G.00032476* were significantly associated with almost all flowering phenology-related traits (Supplementary Data Table S8). Furthermore, 018883F:52624 (*P* = 6.509548E−04) was located in the intergenic region of *psu.G.00006813* and was significantly associated with DLFS (2021) and DLDS (2021). Its homologous sequence is a putative transcriptional activator that may be involved in light control that regulates development. Other loci were significantly associated with multiple flowering phenology traits only in 2021. 033842F:46943 (*P* = 2.073275E−04), was annotated to *psu.G.00010910*. Its homologous genes may prevent the early activation of the vegetative to reproductive transition by regulating flowering. 050563F:22105 (*P* = 1.871660E−02) was annotated to *psu.G.00014617*, encoding a protein similar to PCFS, which inhibits *FLC*-mediated flowering inhibition by regulating selective processing of *FCA* pre-mRNA, thereby promoting flowering. 153530F:9938 (*P* = 9.100045E−03) was closely associated with TCE (2020–21), TBS (2020–21), TIF (2020–21), THO (2020–21), TFB (2020–21), TID (2020–21), TDS (2020), TEFS (2020–21), TLFS (2020–21), TEDS (2020–21), and TLDS (2020–21), and 153530F:9897 (*P* = 3.176314E−02) was closely associated with TCE (2020–21), TBS (2020–21), TIF (2020–21), THO (2020–21), TFB (2020–21), TID (2020–21), TDS (2020), TEFS (2020–21), TLFS (2020–21), TEDS (2020–21), and TLDS (2020). Both were annotated to *psu.G.00027564*, whose homologous gene mediates the suspension of pollen tube growth during fertilization, thus ensuring reproductive isolation barriers. The other homologous genes are mainly involved in the regulation of enzyme activity in important processes of life. qRT–PCR results showed that the average expression levels of 16 genes could be divided into three groups; the average expression levels of *psu.G.00006813*, *psu.G.00010910*, *psu.G.00015854*, *psu.G.00019234*, and *psu.G.00027564* were lower in MU than in FD and LH (Fig. 6Bb). These results indicated that *psu.G.00006813*, *psu.G.00010910*, *psu.G.00015854*, *psu.G.00019234*, and *psu.G*.00027564 were also candidate genes for the control of flowering in this tree peony population.

To compare flowering phenology with genotype at these candidate genes, individual plants from the GWAS populations were grouped by their genotypes at *psu.G.00006813* (018883F:52624), *psu.G.00010910* (033842F:46943), *psu.G.00015854* (056899F:12609), *psu.G.00019234* (076608F:19232 and 076608F:19172), and *psu.G.00027564* (153530F:9938 and 153530F:9897). At 018883F:52624, 033842F:46943, and 056899F:12609, the GWAS population was divided into two genotypes, and most trait values were higher for individuals with the homozygous genotype (018883F:52624 GG, 033842F:46943 CC, and 056899F:12609 AA) than for those with the heterozygous variant genotype (018883F:52624 GA, 033842F:46943 CT, and 056899F:12609 AG) (*P* < .05) (Fig. 6Ca–c). In the GWAS population, there were three genotypes of the SNPs 076608F:19232, 076608F:19172, 153530F:9938, and 153530F:9897: the main homozygous genotypes (076608F:19232 CC, 076608F:19172 GG, 153530F:9938 AA, and 153530F:9897 CC), homozygous variant genotypes (076608F:19232 TT, 076608F:19172 AA, 153530F:9938 GG, and 153530F:9897 TT), and heterozygous variant genotypes (076608F:19232 CT, 076608F:19172 GA, 153530F:9938 AG, and 153530F:9897 CT) (Fig. 6Cd–g). The flowering phenology traits of individuals with homozygous alleles at 076608F:19232 and 076608F:19172 were significantly higher than those of individuals with heterozygous genotypes in different years (*P* < .05) (Fig. 6Cd and e). However, for 153530F:9938 and 153530F:9897 the flowering phenology duration-related traits of individuals with homozygous alleles were significantly higher than those of individuals with heterozygous allele genotypes in different years (*P* < .05). The flowering phenology timing-related traits of individuals with heterozygous variant genotypes were significantly higher than those of individuals with homozygous allelic genotypes (*P* < .05) (Fig. 6Cf and g).

### Identification of candidate genes for floral agronomic traits based on GWAS

GWAS was used to analyze four floral agronomic traits (NF, FH, FD, and PL) of the 451 tree peony varieties (Supplementary Data Tables S12 and S13, Supplementary Data Fig. S8). From 2019 to 2021, 414, 557, and 533 SNPs were significantly associated with the four traits (*P* < 9.34E−04), and 61 SNPs were repeatedly detected in at least 2 years. There were 439, 410, 351, and 270 SNPs associated with NF, FH, FD, and PL (*P* < .001), respectively, and these SNPs were annotated to 151, 104, 126, and 99 genes. Seventeen (NF), 2 (FH), 3 (FD), and 4 (PL) of these genes were identified in at least 2 years (Supplementary Data Table S5).

NF is an important trait of tree peony, and an SNP highly associated with NF was detected in 2019 and 2020. 003970F:167592 (*P* = 6.692498E−04) corresponded to *psu.G.00001860*, a homolog of *A. thaliana* that encodes histone H2B1 (Supplementary Data Fig. S8Aa and b, Supplementary Data Tables S12 and S13). 022030F:57770 (*P* = 3.225309E−02) was associated with NF in 2020 and 2021 and was annotated to *psu.G.00007744*. This gene is homologous to *PGK1* in *A. thaliana*, whose protein is involved in the Calvin cycle, a central pathway of carbohydrate biosynthesis (Supplementary Data Fig. S8Ab and c, Supplementary Data Tables S12 and S13). In 2019 and 2021, 054436F:26665 (*P* = 6.947222E−01) was associated with NF and was located in the exonic region of *psu.G.00015402*, annotated as a heat shock 70-kDa protein. 123085F:9946 (*P* = 7.686874E−04) corresponded to *psu.G.00024852*, which was annotated as a pentatricopeptide repeat-containing protein (Supplementary Data Fig. S8Aa and c, Supplementary Data Table S12). Several additional SNPs associated with NF were identified in at least 2 years: 031300F:31497 (*P* = 2.040745E−01), corresponding to *psu.G.00010275*; 040525F:28828 (*P* = 1.446972E−02), corresponding to *psu.G.00012484*; and 001375F:165072 (*P* = 3.669752E−04), corresponding to *psu.G.00000739* and *psu.G.00000740*. These genes did not have functional annotations (Supplementary Data Fig. S8A, Supplementary Data Table S13).

039452F:85418 (*P* = 2.610211E−01, detected in 2019) and 039452F:85497 (*P* = 2.923983E−04, detected in 2021) were significantly associated with FH and were annotated to *psu.G.00012246*. Its homologous gene encodes a transaminase that inhibits stem branching (Supplementary Data Fig. S8Ba and c, Supplementary Data Table S12). An SNP (069898F:1423, *P* = 4.054218E−01) significantly associated with FH in 2020 was found upstream of a gene annotated as methionine adenosine transferase (Supplementary Data Fig. S8Bb, Supplementary Data Table S12). The SNP 112201F:22843 (*P* = 6.215242E−05) for FH in 2021 was detected in the exonic region of *psu.G.00023736*, annotated as an *O*-aminobenzoic phosphoribose transferase involved in l-tryptophan biosynthesis (Supplementary Data Fig. S8Bc, Supplementary Data Table S12). In 2019, the synonymous SNP 196127F:5101 (*P* = 2.594032E−01) was also found to be associated with FH, but it had no corresponding annotation information (Supplementary Data Fig. S8Ba, Supplementary Data Table S12).

A significant peak at 023234F:9925 (*P* = 7.434257E−01) appeared on the GWAS Manhattan plots for FD in 2020 and 2021. This locus corresponded to *psu.G.00008117*, which had no specific annotation information (Supplementary Data Fig. S8Cb and c, Supplementary Data Tables S12 and S13). In addition, the non-synonymous SNP 222627F:15902 (*P* = 8.236864EA, significant peak at 023234F:992501) was associated with FD in 2021 and corresponded to *psu.G.00031965*, which is a possible transcriptional regulator (Supplementary Data Fig. S8Cc, Supplementary Data Table S12). Similarly, 035125F:31609 (*P* = 5.480356EA, significant peak at 023234F:992504) was detected in both 2020 and 2021 and was significantly associated with PL. This locus corresponded to *psu.G.00011223*, which was predicted to encode a protein containing a zinc finger CCCH domain (Supplementary Data Fig. S8D, Supplementary Data Tables S12 and S13). 047095F:46670 (*P* = 5.425190EA, significant peak at 023234F:992506) corresponded to *psu.G.00013920*, which may participate in the splicing of precursor mRNA (Supplementary Data Fig. S8D, Supplementary Data Tables S12 and S13).

Interestingly, some SNPs were associated with more than one trait in multiple years and may therefore be loci that regulate multiple traits (Supplementary Data Table S8). 054011F:52593 (*P* = 1.460466EA, significant peak at 023234F:992501) was repeatedly detected for NF and PL in different years. This locus corresponds to *psu.G.00015304* and is annotated as a possible motility protein with terminal orientation. 139798F:23560 (*P* = 9.594038−EA, significant peak at 023234F:992504) was significantly associated with NF in 2019 and PL in 2021 and was located upstream of *psu.G.00026408*. This gene was annotated as citrate synthase, which is present in almost all cells with oxidative metabolism capacity. 028091F:77862 (*P* = 9.326180E−04), located in an intronic region, was associated with FH in 2020 and FD in 2021; this locus corresponded to *psu.G.00009422*, a homolog of *SYP121*, which encodes a vesicle transport protein in the secretory pathway (Supplementary Data Table S8).

### Cumulative effect of increasing-effect alleles on flowering phenology traits and floral agronomic traits

To further investigate the effects of combined alleles on FDT and NF or FD, the number of increasing-effect alleles in each accession was determined. The number of increasing-effect alleles possessed by each variety ranged from one to seven, and most had more than three (Supplementary Data Fig. S9A, Supplementary Data Table S14). Significant correlations were observed between FDT and number of increasing-effect alleles, with *r*^2^ = .76 and 0.78 (*P* < .001) for 2019 and 2021, respectively (Supplementary Data Fig. S9Ba and b). Linear regressions were performed using the 3-year mean values to further investigate the relationship between FDT and number of increasing-effect alleles. There was a significant linear relationship between FDT and number of increasing-effect alleles, with a regression slope of 0.25 (*r*^2^ = .85, *P* < .001) (Supplementary Data Fig. S9Bc). There were also clear cumulative effects of allele number on NF and FD (*P* < .001). Linear regression slopes for NF-2020, NF-2021, and 3-year mean values versus the number of increasing-effect alleles were 0.82 (*r*^2^ = .74), 0.81 (*r*^2^ = .55), and 0.74 (*r*^2^ = .61), respectively (Supplementary Data Fig. S9C). The linear regression slope between FD in 2020 and the number of increasing-effect alleles was .18 (*r*^2^ = .84), and the benefits tended to stabilize when the number of increasing-effect alleles increased to four (Supplementary Data Fig. S9D).

## Discussion

Phenological observations and phenotypic selection have always been important approaches to studying the biological and ecological characteristics of plants [45, 46]. In this study, we found that the flowering duration of tree peony was relatively concentrated, mainly occurring in April. However, the specific occurrence times and duration of phenophase varied greatly among different varieties. Flowering phenology also differed among years, consistent with flowering phenology reports for other plants [47]. This is because flowering phenology is not only controlled by the internal environment and genetic information but also influenced by the external climate [48]. Previous reports revealed that the variationof 22 quantitative characters in the association analysis population of seedling population of *Paeonia rockii* hybrids was highly significant (*P* < .01), and the CV was 10%–30% [49]. Similarly, 462 individuals of *P. rockii* in the associated population were evaluated according to 12 traits. The phenotypic variation range of all measured traits was wide, and the CV of petal number was the highest (112.10%), while the CV of petal shape was only 9.52% [50]. The heterosis analysis of 20 phenotypic characters in the *F*_1_ population of tree peony hybrid showed that each phenotypic character was widely segregated in the *F*_1_ population, and the CV ranged from 11.03% to 63.49% [51]. Moreover, the phenotypic diversity of nine natural populations of *Paeonia delavayi* was studied by using 31 morphological indexes. It was found that the CV ranged from 3.22% to 76.12%, and the average CV among populations was 25.24% [52]. The degree of variation in 23 flowering phenology-related traits and 7 ornamental traits of tree peony observed in this study differed from a previous report, perhaps because of the differences in population size, plant materials, cultivar-group construction, and modulation traits. We also found highly significant correlations among flowering phenology phenotypes, meaning that the interactions of these traits must be considered during selection and that enhancement of one trait will lead to improvement in other traits [53].

GWAS is an effective way to discover the genetic basis of complex traits. With the development of genomics research, GWAS can easily and directly explain correlations between phenotypes and genes, and this method has therefore been widely used for screening and identification of major genes for economically important agricultural traits [19, 54–56]. Information obtained by GWAS can be used to develop functional molecular markers for marker-assisted breeding [57–59]. The choice of materials for GWAS is very important, and materials with greater regional differences and richer phenotypic differences are more conducive to association analysis [60]. The population used in this study consisted of 451 ornamental tree peony varieties; it had abundant phenotypic variation, providing a good basis for GWAS analysis. Owing to the complexity of population structure and genetic relationships among accessions, false positives between markers and traits occasionally occur in GWAS analysis; researchers have therefore developed a variety of GWAS analysis models [61, 62], and selection of an appropriate model is crucial. In this study, we used an MLM with population structure as a fixed effect and related-relationship matrix as a random effect for association analysis, and a Bonferroni correction threshold was used to reduce the probability of false positives. The observed *P* values of the MLM analysis results fit well with the expected *P* values in the association analysis of various traits (Supplementary Data Figs S4–S7), indicating that the MLM model was suitable for this study.

Reduced-representation genome sequencing (RRGS) is a relatively efficient method for population analysis [63]. The existing methods mainly include restriction fragment length polymorphism (RFLP) [64] and specific locus amplified fragment sequencing (SLAF-seq) [65, 66]. The new genotyping method based on sequencing by GBS has been gradually applied to many kinds of plant studies due to its advantages of speed, simplicity, and low cost [67, 68]. At present, this method for GWAS is employed for many plants with large genomes. In CIMMYT’s semiarid wheat breeding program, 41 371 SNPs were identified by GBS in 254 advanced breeding lines, and multiple SNPs in the same tag were removed; 34 749 SNPs were finally retained for subsequent analysis [69]. Similarly, in the elite yield trials evaluation experiment of four groups of wheat breeding lines including this population in 2019, 78 606 markers were strictly filtered, resulting in 16 072 GBS markers showing the effect of high coverage on trait predictability for subsequent trials [70]. The high-quality SNPs obtained from 230 garlic germplasms screened by GBS technology were sequenced to infer population structure, and four main groups were found in garlic germplasm [71]. GBS analysis of 190 young pepper leaves produced only 4083 effective SNPs, which elucidated the origin of Spanish regional varieties and the path of pepper evolution [72]. At present, the obtained tree peony genome is up to 13.79 Gb. We finally screened 107 050 high-quality SNPs for subsequent GWAS analysis using GBS technology, which is comparable to the studies in wheat and garlic or even better than them considering the number of identified SNPs over the size of the genome.

Flowering time is a complex environmental response trait with important implications for plant adaptation, crop yield, and reproductive isolation [73, 74]. Therefore, it is very important to explore the genetic variation that regulates the flowering network. At present, GWAS has revealed the molecular basis of flowering time, cooling requirement, and other phenological traits in *Arabidopsis* [31], *Prunus persica* [75, 76], *Populus simonii* [73], and *B. napus* [26, 27]. It has also demonstrated that flowering phenology traits are controlled by multiple genes. Using a large tree peony germplasm collection, we identified SNPs that were closely associated with 23 flowering phenology traits and 4 floral agronomic traits, revealing strong interactions between genotypes and phenotypes. We used 23 flowering phenology traits to describe flowering events and capture the dynamic process of tree peony flowering. We observed strong consistency between flowering phenology timing-related and duration-related traits, and we identified several SNPs significantly associated with multiple flowering events, highlighting the effectiveness of GWAS for studying the regulation of flowering in tree peony.

GWAS is not yet well established in tree peony despite the availability of stable and in-depth sequencing platforms, as high-quality genome sequences for tree peony have only recently been developed [3]. Based on the tree peony genome data, GWAS was used to identify SNP markers related to flowering phenology traits, but it did not detect many flowering QTLs reported by others. However, some SNPs associated with known flowering regulation genes were found among the identified SNPs. A significant SNP was found in 2020 in *PoFY* (Fig. 4), which acts to correctly locate the polyadenylation of *FLC* to control flowering time. As an inhibitor of *FLC*, *FY* can interact with *FCA* to inhibit the accumulation of *FLC* [77–79]. Research also suggests that *FY* has a dual role in the regulation of flowering time, as a hypomorphic allele of *FY* (fy-5) can cause later flowering by activating *FLC* expression [77]. In addition, five important genes were found to be significantly associated with several flowering phenology traits (Fig. 6). *FER*, the homologous gene of *psu.G.00027564*, can ensure reproductive isolation barriers by regulating the subcellular polarization of pollen tube *MLO7* perception in the female gametophyte to stop compatible pollen tube growth [80, 81]. Subsequently, Yu *et al*. [82] found that *FER* activated ABI2 phosphatase to inhibit abscisic acid (ABA) signaling and suggested that it was a positive regulator of growth. *Psu.G.00006813* may be a *MIK2* homolog involved in pollen tube perception of female signals [83]. *psu.G.00010910*, an *RLT* homolog encoding homeobox-DDT domain protein, is a key gene related to *FT*, *SEP1*, *SEP3*, *AGL8*/*FUL*, *SOC1*, and *FLC*, and is a transcription regulator required to maintain plant nutrition [84]. The homologous gene of *psu.G.00015854*, a member of the ABC Transporter G family, is involved in the intercellular and intracellular ABA signaling pathways leading to stomatal closure, thus conferring drought tolerance [85]. Studies have shown that it can function as a pump and have some resistance to the terpene sclareol [86]. *psu.G.00019234* was homologous to *TOM40*, which is involved in pore formation [87]. In addition, the remaining SNPs that were not yet identified may be novel loci related to flowering phenology and flowering time, and they should be validated by other methods in future studies.

NF, FH, FD, and PL are key traits that determine the ornamental value of tree peony. A number of SNPs with significant associations with NF were identified (Supplementary Data Fig. S8A, Supplementary Data Table S12), including 004142F:147789 (*psu.G.00001944*), 012558F:33747 (*psu.G.00004748*), and 102174F:18598 (*psu.G.00022627*). They were annotated as homologs of *PK1*, *M3KE1*, and *RPL27AB*, respectively, which are mainly involved in cell elongation during pollen development [88] and flower development [89]. An SNP (052652F:40803) closely related to FD corresponded to *psu.G.00015020* (*PI206*) (Supplementary Data Fig. S8C, Supplementary Data Table S12), and most studies have shown that this gene can play a central role in plant secondary metabolism [90]. Several SNPs associated with FH were annotated in members of the *LAC* family, including *psu.G.00002202* (*LAC9*), *psu.G.00002203* (*LAC5*), and *psu.G.00013272* (*LAC17*) (Supplementary Data Fig. S8B, Supplementary Data Table S12). These genes play important roles in degradation of lignin and detoxification of lignin-derived products [24, 91, 92]. During 2 years of PL observations, 047095F:46670 was identified in *psu.G.00013920* (Supplementary Data Fig. S8D, Supplementary Data Tables S12 and S13), which is highly homologous to *PRP31*, a gene whose product participates in the splicing of precursor mRNA under low temperatures and during the stress response [93].

Although many SNPs were detected in this study, most were not associated with any annotated genes. The biological functions of these unknown SNPs deserve further study. In conclusion, the identification of SNPs associated with flowering phenology and other phenotypic traits provides new insights into the genetic mechanism of flowering period regulation in tree peony and provides opportunities for improving its ornamental performance.

## Materials and methods

### Plant materials

The research project was carried out at the tree peony germplasm resources nursery of Henan University of Science and Technology (112°28′36.34″ E, 34°39′30.34″ N) from 2019 to 2021. A total of 596 tree peony varieties from the nursery were evaluated (Supplementary Data Table S1).

### Phenotype measurements

Based on the recording standard for tree peony phenology, reproductive growth was observed from the bud enlargement stage to the petal decay stage. Flowering phenological characteristics were recorded from 9:00–14:00 in 2020 and 2021 and included the timing and duration of the color-exposure stage (CE), blooming stage (BS), initial flowering stage (IF), half opening stage (HO), full blooming stage (FB), initial decay stage (ID), decay stage (DS), early full blooming stage (EFS), late full blooming stage (LFS), early decay stage (EDS), and late decay stage (LDS) [94, 95]. Flowering phenology observations were made daily and recorded using the universal Gregorian calendar date. The day when the first flower bud of *P. suffruticosa* ‘Yupantuojin’ (three consecutive years of investigation found that this variety showed the earliest flowering phynotype) began to break was recorded as the first day, and the time of flowering phenology stages for each variety was calculated based on this date. The durations of flowering phenology stages for each variety were calculated based on the bud-break time of the first flower bud for that variety. The flowering duration time (FDT) was also recorded in 2019, 2020, and 2021.

Flower height (FH) and flower diameter (FD) were measured with an accuracy of 0.01 cm on nine flowers from each tree peony variety in 2019, 2020, and 2021. The number of flowers (NF) was also recorded. Pedicel length (PL), plant height (PH), crown diameter (CD), and crown shade size (CSS) were measured in 2020 and 2021.

Descriptive statistics, including the mean, median, and variance, were calculated for all variables. Coefficients of variation (CV = SD/mean × 100) were used as the indicator of variability. Correlations between traits were determined by calculating the Pearson correlation coefficient (*P* ≤ .05) to reveal possible associations among the data. PCA was used to study the relationship between the traits. All statistical analyses were performed using SPSS 19.0 (SPSS, Inc., Chicago, USA) and R v.4.1.3 software.

### Genotyping by sequencing of tree peony cultivars

Fresh, healthy petals from each varieties at FB were collected and immediately frozen in liquid nitrogen before extraction of genomic DNA by using a previously modified cetyltrimethyl ammonium bromide (CTAB) method [4, 96]. GBS libraries were constructed and sequenced for genome sequencing using an Illumina HiSeq PE150 instrument (Illumina Inc., USA) at Novogene Bioinformatics Technology Co., Ltd (Beijing, China). Valid sequences were aligned to the tree peony reference genome (https://db.cngb.org/search/project/CNP0000281) [3] using the Burrows–Wheeler Aligner software (BWA, version 0.7.12) [97]. The Genome Analysis Toolkit (GATK, version 2.4) was used to collect a total of 45 236 236 SNPs. After filtering with the parameters dp4 (depth not less than 4×), Miss0.1 (missing rate not higher than 0.1), and Maf0.05 (minimum allele frequency not less than 0.05), 107 050 high-quality SNPs were obtained for subsequent GWAS.

### Population structure control

The hidden population structure and individual kinship were modified using the MLM in STRUCTURE v2.3.4. Five independent runs were executed for each number of subpopulations (*K*) ranging from 1 to 10. The run-in time and MLM copy count were both set to 10 000 for each run. The distance matrix was calculated by using TreeBest v1.9.2 (http://treesoft.sourceforge.net/treebest.shtml). PCA was performed using GCTA software (https://cnsgenomics.com/software/gcta) and the ggplot2 package of the R v.4.1.3 software. The neighbor-joining method was used to construct phylogenetic tree, and the significance level of the eigenvectors was determined using the Tracy–Widom test.

### Genome-wide association study

GWAS was performed using 451 tree peony varieties and 107 050 high-quality SNPs using GEMMA software (http://www.xzlab.org/software.html) and Bonferroni-corrected test thresholds. MLM was used for trait correlation analysis, with population genetic structure treated as a fixed effect and individual relatedness treated as a random effect, which was corrected for the influences of population structure and individual relatedness:

(1) \begin{equation*} Y=a{X}_1+b{X}_2+c{X}_3+e \end{equation*}

where *Y* is the phenotypic trait, a is the fixed effect indicator matrix, *X*_1_ is the estimated parameter of the fixed effect, b is the index matrix of SNPs, *X*_2_ is the effect of SNPs, c is the index matrix of the random effect, *X*_3_ is the random individual of prediction, and e is the random residual and obeys e ~ (0, δ E2).

Negative log_10_ (*P*) was used as a threshold to assess the significance of associations between SNPs and traits of interest. SNPs with −log_10_ (*P*) > 3 were considered significant. The GWAS results were created in the form of Manhattan and Q–Q plots drawn by the qqman package of the R v.4.1.3 software. Because the tree peony genome is a contig version at present and there is little annotation information, we extended the annotation range of each SNP to the whole contig to search for possible candidate genes.

### Establishment of transient expression system of tree peony

Plant expression vectors pCAMBIA2300 were digested with restriction enzyme KpnI using correctly sequenced positive cloned plasmids as templates. The overexpression vector pCAMBIA2300-*PoFY* was obtained by seamless cloning technology, and the plasmid extracted from the correct bacterial solution was screened and sequenced to transform *Agrobacterium* GV3101. The transformed *A. tumefaciens* harboring pCAMBIA2300 empty vector and pCAMBIA2300-PoFY were grown at 28°C in the lysogeny broth (LB) medium (supplemented with 50 μg·ml^−1^ kanamycin and 20 μg·ml^−1^ rifampin) for 24 hours. The primers used in this study are listed in Supplementary Data Table S15.

The flowers of *P. suffruticosa* ‘Luoyanghong’ (petals purplish red, base with inky purple spots) at BS with 5 cm pedicels were cut off and placed in 40 ml of bacterial suspension solution, and the buds were wrapped with gauze and soaked in 10 ml of bacterial suspension solution. The buds were vacuumized to 0.07 MPa for 30 minutes, and then slowly exhaled. The aspirated petals were rinsed with deionized water to remove excess bacterial suspension solution. The pedicels were kept immersed in the corresponding bacterial suspension solution and cultured in the dark at 8°C for 2–3 days, then transferred to 23°C for 1 day. Subsequently, the fresh cut tree peony flowers were cultured under normal light and sampled every 7 hours. The expression levels of target genes and the flowering time of fresh cut flowers were determined and compared with the control.

Similarly, fresh petals and disks (diameter 1.50 cm) were collected from *P. suffruticosa* ‘Luoyanghong’ at HO, wrapped in gauze, and immersed in the same bacterial suspension solution as the above fresh cut flowers. The tree peony petals or disks were infiltrated under a vacuum of 0.07 MPa for 10–15 minutes. After releasing the vacuum, the petals and disks were rinsed in ddH_2_O and placed in sterile petri dishes (9 cm in diameter) with 5–10 ml of ddH_2_O. In order to achieve sufficient infection the samples with bacterial suspension solution, petals or disks were subjected to the same infection treatment as described above for the cut flowers before phenotypic observation. At the end of bacterial infection, the tree peony petals or disks were dehydrated for 12 hours and then rehydrated for 12 hours. We measured the length, width, and fresh weight of all petals or disks every 3 hours, and the expression level of the target gene was determined using qRT–PCR.

### Expression analysis of candidate genes

qRT–PCR was performed using the SYBR^®^ Green *Pro Taq* HS premixed qPCR Kit II (Accurate Biology, China) on a CFX Real-Time System (Bio-Rad). Total RNA was extracted at different flower development stages (CE, BS, IF, HO, FB, ID, and DS) from the early-flowering mutant and wild *P. ostii* ‘Fengdan’, and *P. suffruticosa* ‘Lianhe’ (MU, FD, and LH for short). qRT–PCR primers were designed using Primer Premier 5.0 (Supplementary Data Table S15). The 20-μl qRT–PCR reaction contained <100 ng template, 10 μl 2 × SYBR^®^ Green *Pro Taq*HS Premix II, 0.8 μl of each forward and reverse primer, and 7.4 μl ddH_2_O. *P. ostii* ‘Fengdan’ *EF1-α* was used as a reference gene to determine the relative expression of the target genes. The relative expression levels of target genes were calculated using the 2^-ΔΔCt^ method. Data obtained from four independent RNA extractions were used for statistical analysis.

## Acknowledgements

This research was supported by the National Natural Science Foundation of China (U1804233), the Innovation Scientists and Technicians Troop Construction Projects of Henan Province (202101510003), and the Outstanding Youth Fund of the Natural Science Foundation of Henan Province (202300410119).

## Author contributions

Y.Y.L. contributed to material preparation, phenotype investigation, methodology, data curation, writing, reviewing, and editing. L.L.G. contributed to material preparation, methodology, data curation, writing, reviewing, and editing. Z.Y.W. contributed to the phenotype investigation. D.H.Z. and D.L.G. contributed to data curation. W.L.Y. contributed to manuscript reviewing and editing. J.E.C. contributed to manuscript reviewing and language embellishment. X.G.H. contributed to the conceptualization, experimental design, supervision and funding acquisition.

## Data availability

Raw sequencing reads relative to the GBS experiment of all tree peony germplasm resources reported in this study have been deposited into the Genome Sequence Archive (GSA) under the accession number CRA007681.

## Conflicts of interest

The authors declare that they have no competing interests.

## Supplementary data

Supplementary data are available at *Horticulture Research* online.

## Supplementary Material

Web_Material_uhac263
