# Supplementary material for: Genome-wide association study of 23 flowering phenology traits and 4 floral agronomic traits in tree peony (Paeonia section Moutan DC.) reveals five genes known to regulate flowering time
Source: Hortic Res. 2022 Dec 2;10(2):uhac263. doi: 10.1093/hr/uhac263 (PMC9926158; doi:10.1093/hr/uhac263)
Supplement: Web_Material_uhac263 [file web_material_uhac263.zip › GWAS-Supplementary Figure.docx]

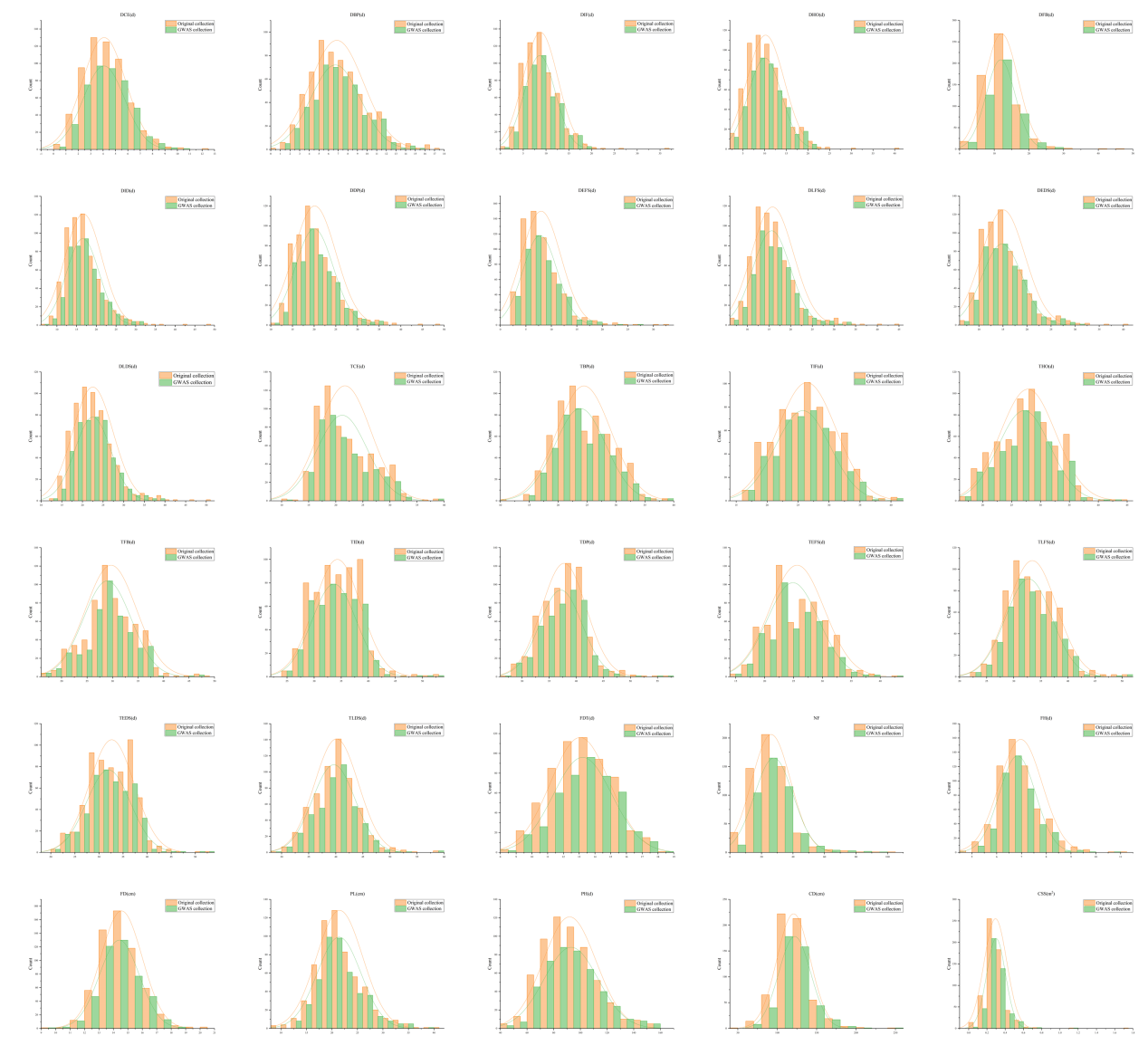


Figure S1 Frequency distribution of accessions in the original and GWAS varieties.
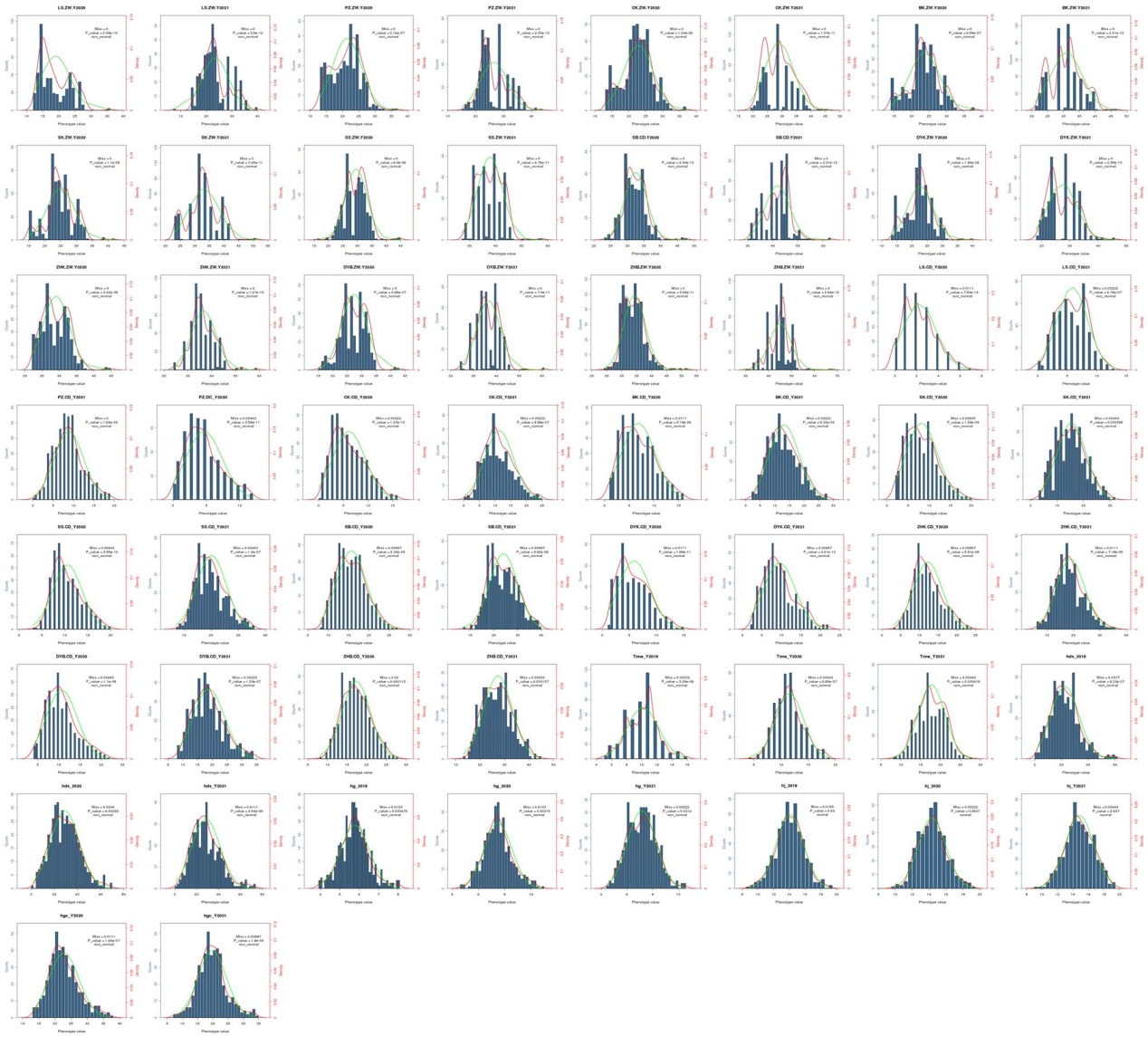
Figure S2 Frequency distribution of different traits in tree peony.


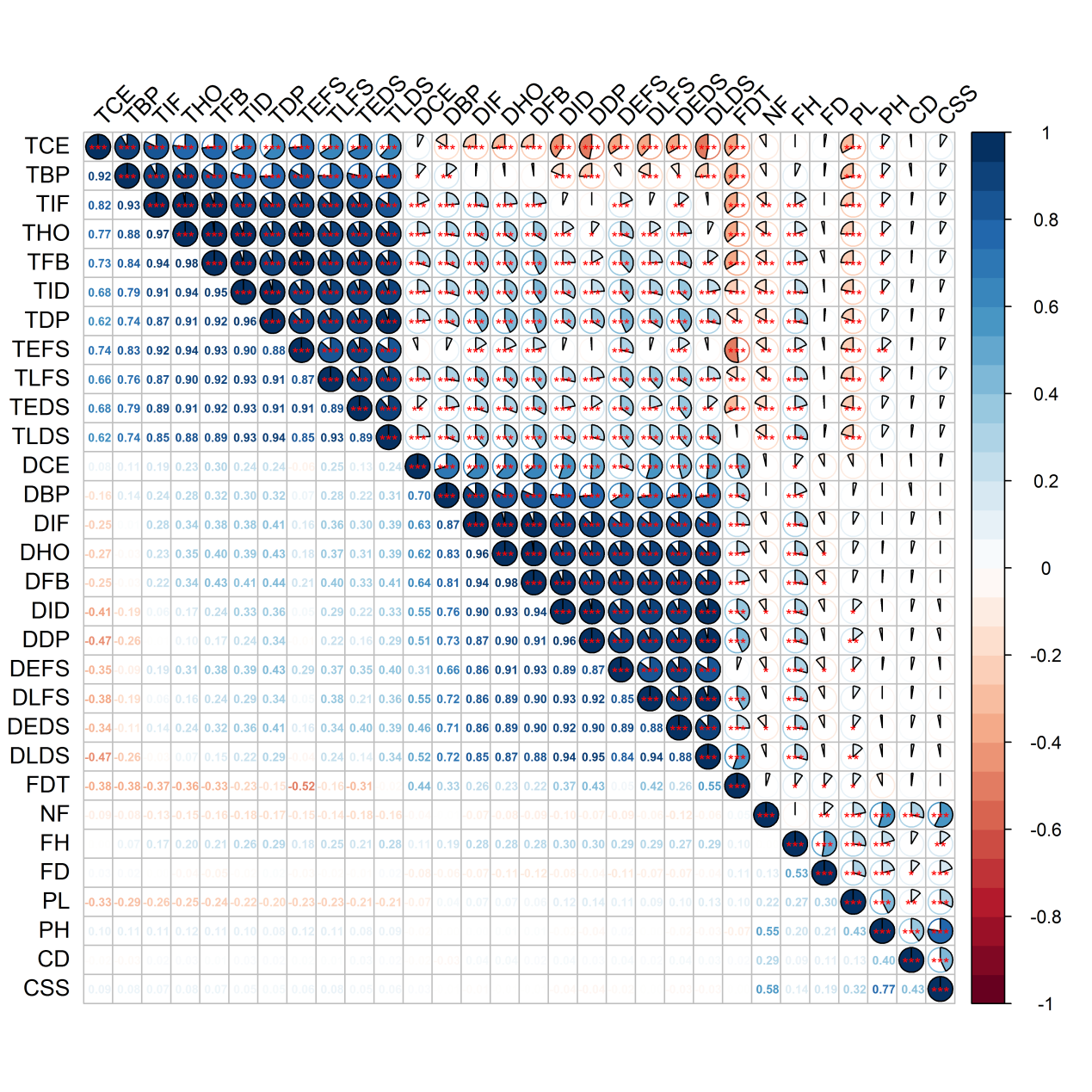


Figure S3 Pearson correlation coefficients calculated among the traits of 451 GWAS varieties evaluated in the study.


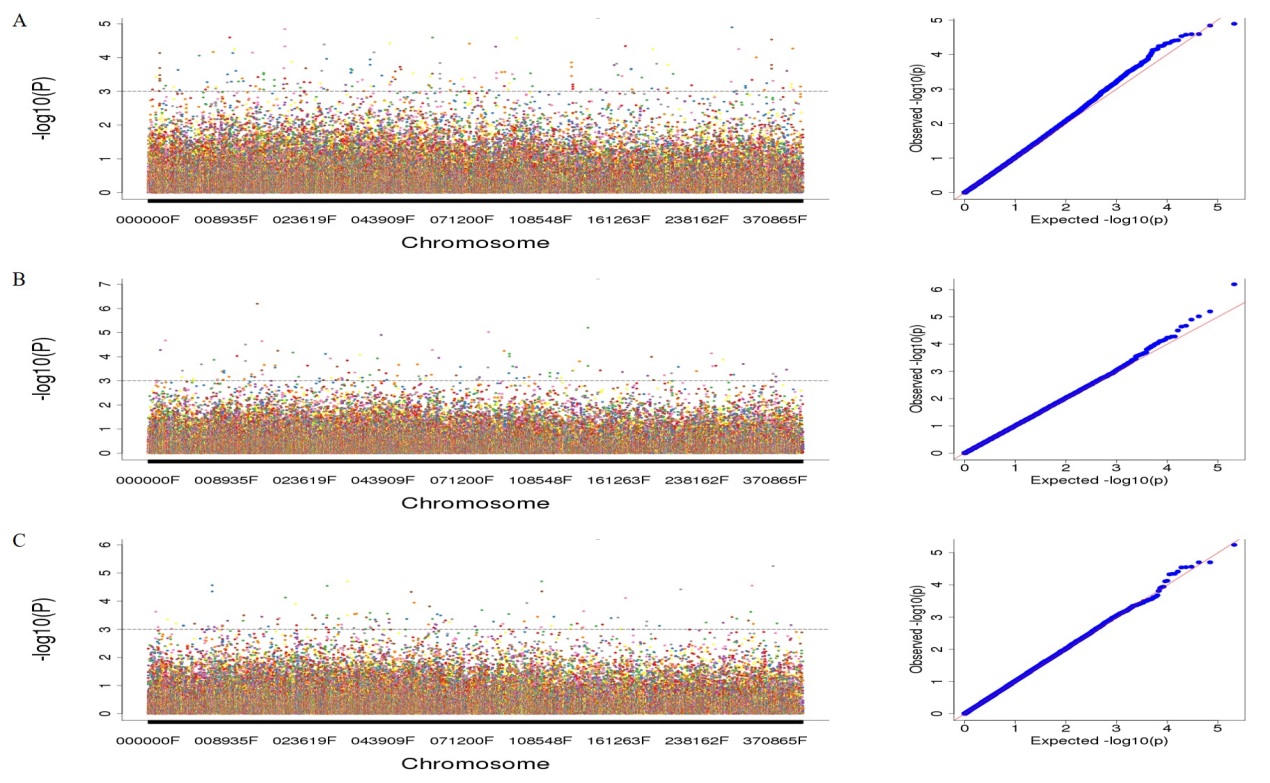


Figure S4 Manhattan plot of -log_10_ (*P*) and Quantile-Quantile plot for SNP-wise associations with FDT in 2019 (A), 2020 (B), and 2021 (C). The gray dotted depicts the significance threshold (log_10_ *P* =3)


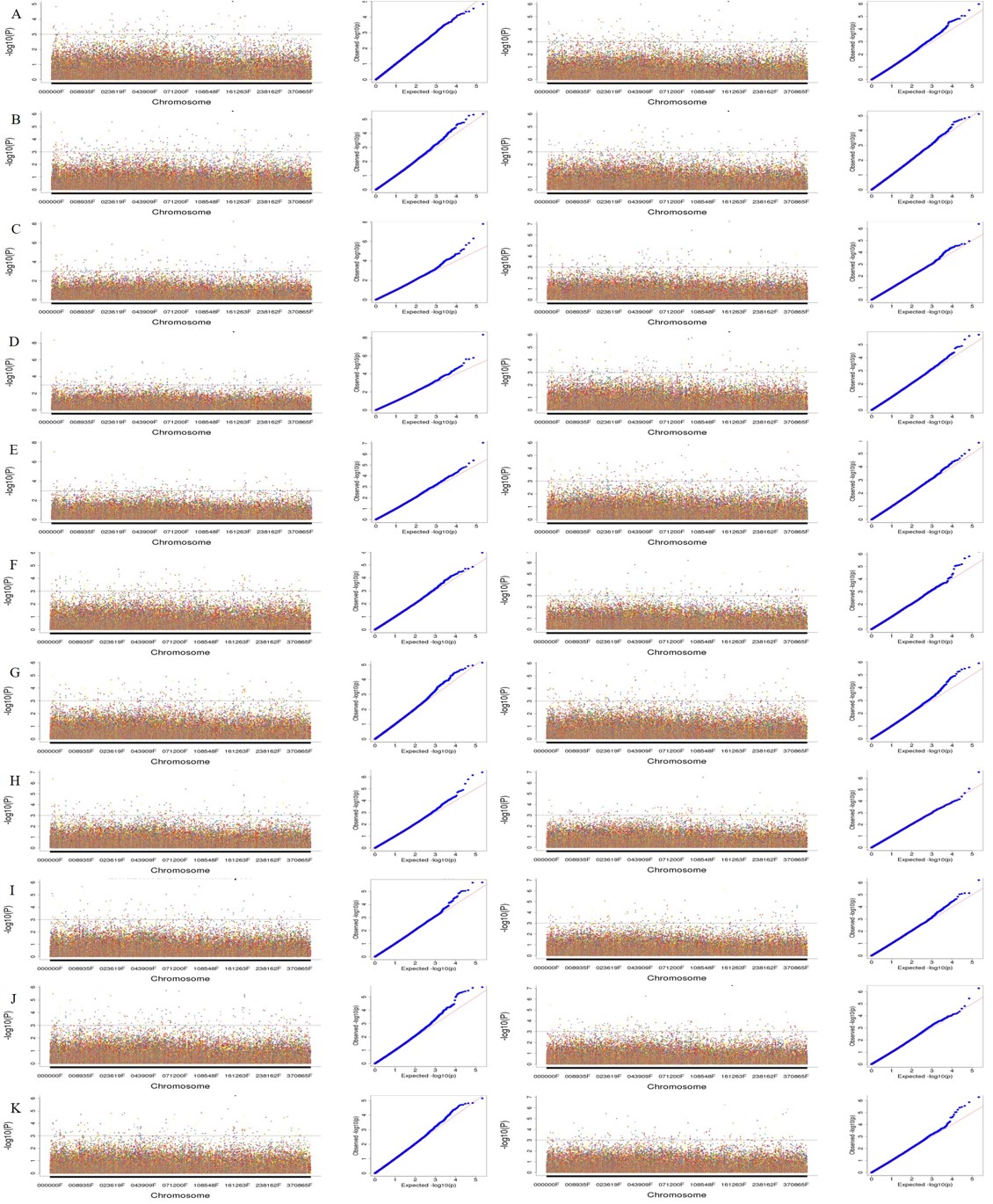


Figure S5 Manhattan plot of -log_10_ (*P*) and Quantile-Quantile plot for SNP-wise associations with TCE (A), TBS (B), TIF (C), THO (D), TFB (E), TID (F), TDS (G), TEFS (H), TLFS (I), TEDS (J), and TLDS (K) in 2020 (a) and 2021 (b). The gray dotted depicts the significance threshold (log_10_ *P* =3)


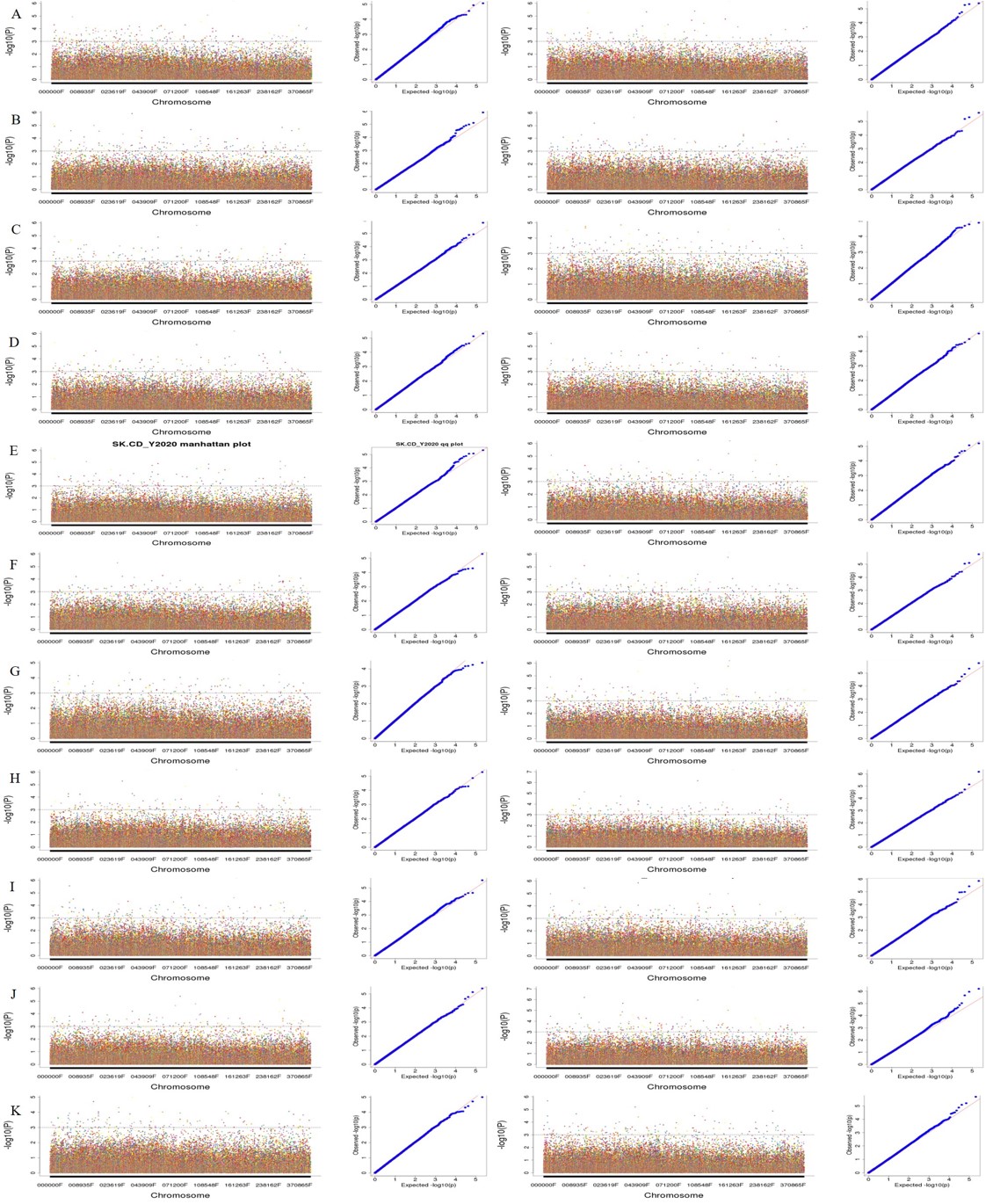
Figure S6 Manhattan plot of -log_10_ (*P*) and Quantile-Quantile plot for SNP-wise associations with DCE (A), DBS (B), DIF (C), DHO (D), DFB (E), DID (F), DDS (G), DEFS (H), DLFS (I), DEDS (J), and DLDS (K) in 2020 (a) and 2021 (b). The gray dotted depicts the significance threshold (log_10_ *P* =3)


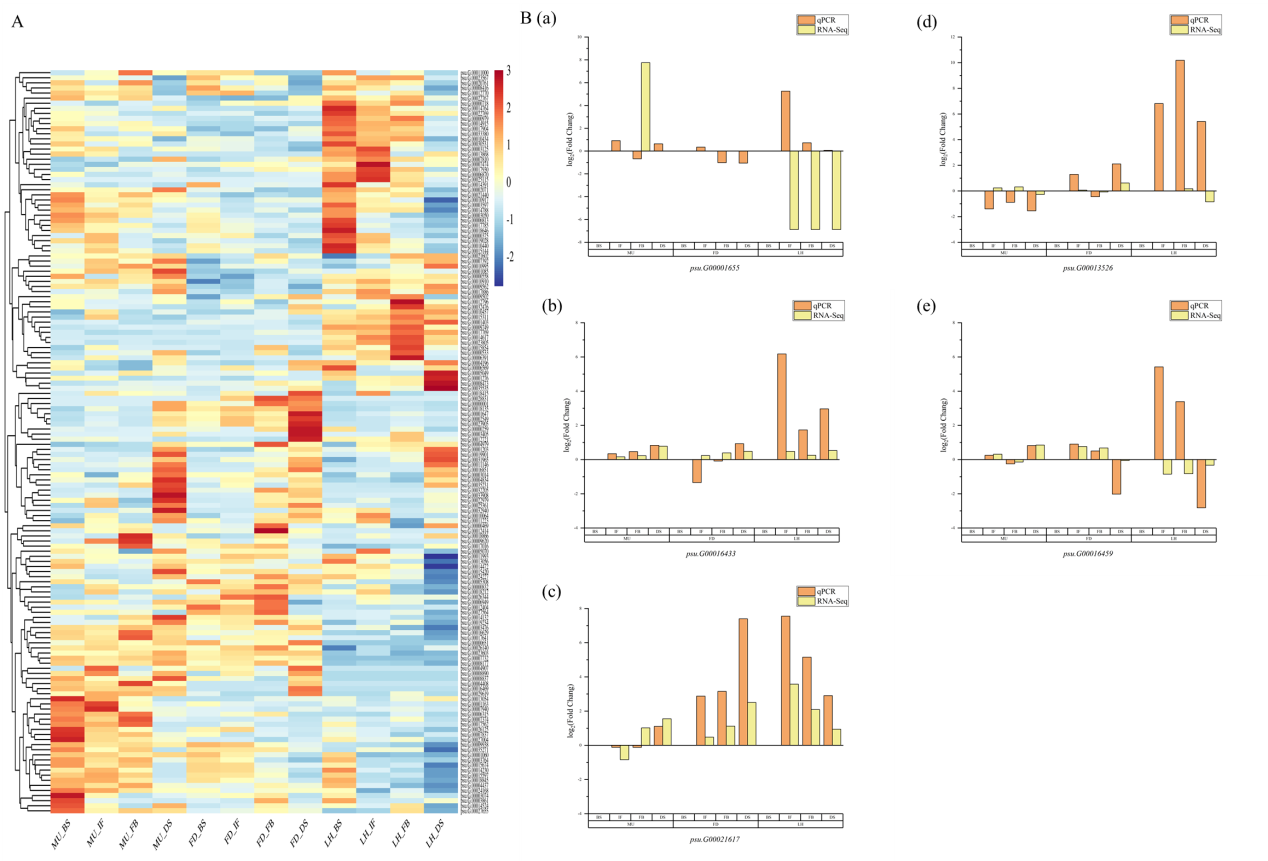


Figure S7 Distribution of differential gene expression levels. A: Heatmaps of differential gene expression. B: RNA-Seq and qPCR results were verified.


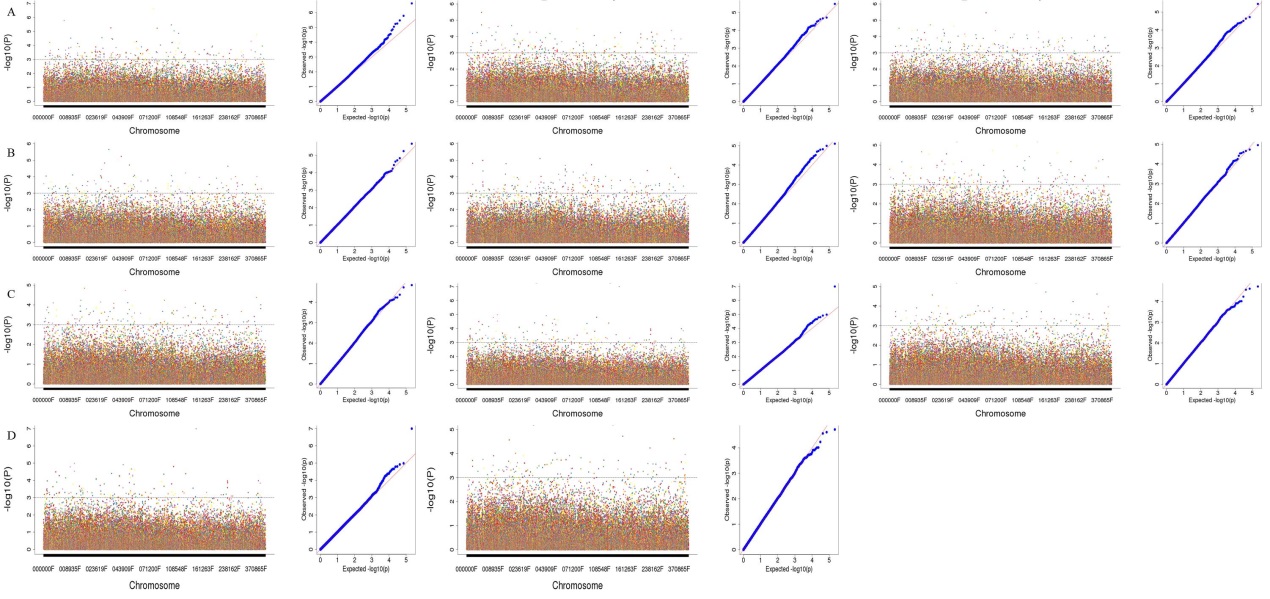


Figure S8 Manhattan plot of -log_10_ (*P*) and Quantile-Quantile plot for SNP-wise associations with NF (A), FH (B), FD (C) in 2019 (a), 2020 (b)and 2021 (c). And PL (D) in 2020 (a) and 2021 (b). The gray dotted depicts the significance threshold (log_10_ *P* =3)


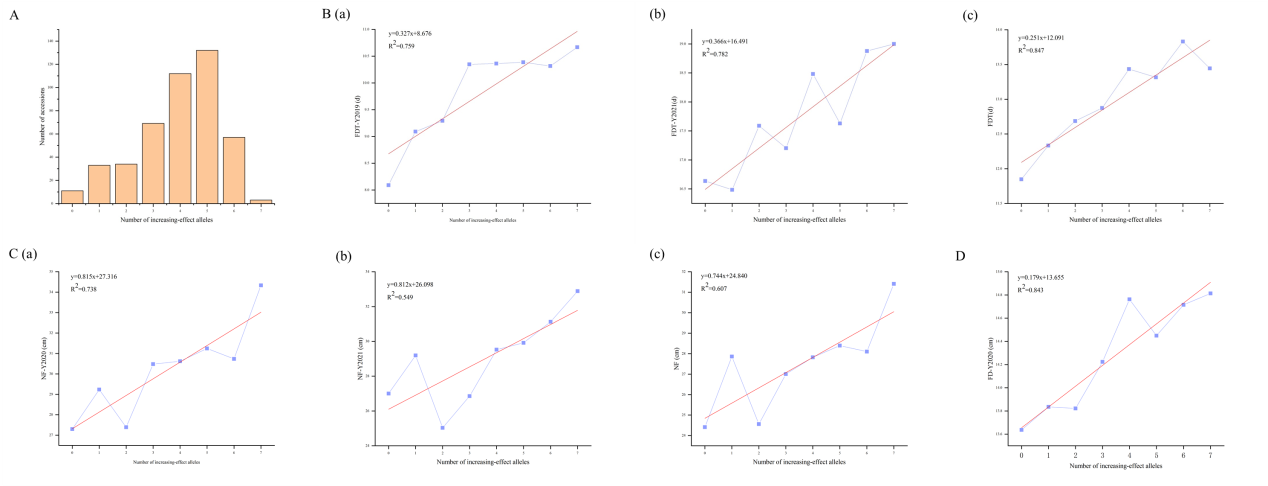


**Figure S9** Cumulative effects of increasing-effect alleles on FDT, NF and FD. The x-axis shows the number of increasing-effect alleles. Points represent the mean values. Linear regressions were performed to investigate the relationships between FDT, NF, and FD and number of increasing-effect alleles in 451 tree peony varieties.
